# Editorial: Olfactory neuroepithelium-derived cellular models to study neurological and psychiatric disorders

**DOI:** 10.3389/fnins.2023.1203466

**Published:** 2023-05-09

**Authors:** Kun Yang, Oleg V. Evgrafov

**Affiliations:** ^1^Department of Psychiatry, Johns Hopkins University, Baltimore, MD, United States; ^2^Department of Cell Biology, SUNY Downstate Health Sciences University, Brooklyn, NY, United States

**Keywords:** olfactory neuroepithelium, neurodegenerative disorders, psychiatric disorders, neurodevelopment, mesenchymal cells, cellular model, drug targets

Neurodevelopment is a spatially and temporally regulated process that occurs mostly during the embryonic and fetal stages. Given the sophistication of the nervous system, particularly the brain, we can only imagine the complexities of the regulation of neurodevelopment. Many human diseases are rooted, or likely rooted, in this period, including some neurological and psychiatric disorders. Although genotyping or sequencing can provide us with genetic information about each patient, we are still a long way from understanding how specific combinations of SNPs and mutations translate into changes in the functionality of different cell types involved in neurodevelopment. While we cannot go back in time to study the embryonic and fetal development of current patients, there is an option to investigate the specifics of neurodevelopment that are ongoing throughout adulthood in the olfactory neuroepithelium (ON) to replenish short-lived olfactory neurons (Graziadei and Graziadei, 1979). ON has been successfully utilized to unveil the neuronal signatures of neurological and psychiatric disorders such as schizophrenia, first-episode psychosis, and bipolar disorder (Mackay-Sim, 2012; Kano et al., 2013; Mor et al., 2013; Lavoie et al., 2017; Rhie et al., 2018; Evgrafov et al., 2020; Namkung et al., 2023). Moreover, constant neuronal loss in the ON may share mechanisms with neuronal degeneration in the human brain, thus making the ON an attractive candidate for modeling neurodegenerative disorders such as Alzheimer's (Rantanen et al., 2022).

The relatedness between the brain and olfactory neuroepithelium may not be limited to the similarity of cell types at early stages of development, but it could also be maintained by continuous interaction through the lymphatic vasculature (Palominos et al.), contributing to the concordance in cell function properties between the ON and the brain. The potential link between the olfactory system and higher brain functions has also been investigated in psychotic disorders and mouse models (Etyemez et al., 2021, 2022; Hasegawa et al., 2021, 2022; Yang et al., 2021).

In agreement with this notion, olfactory deficits have been consistently reported in patients with neurological and psychiatric disorders, such as schizophrenia (Moberg et al., 2014), first-episode psychosis (Kamath et al., 2018), and Alzheimer's disease (Murphy, 2019). In this special topic, Fang et al. reported that severe olfactory dysfunction in patients with Parkinson's.

It is easy to speculate that diseases are caused by alterations in the expression profiling of cells of neuronal lineage—such as neural stem cells, neural progenitors, or even immature neurons—which affect the normal process of differentiation. Such a view may not fully describe the mechanism of diseases and could even be largely wrong. The ON represents a whole ecosystem supporting constant neurogenesis, and it includes other cell types supporting and regulating this process. One of such cell types, which can generally be called mesenchymal cells, may play an important role both in neurogenesis and in the etiology of neurological and psychiatric diseases. Such cells in the ON were described by Delorme et al. (2010), who named them “ectoderm-mesenchymal stem cells.” This team presented their new study in this special topic, describing a protocol for an efficient manufacturing process for clinical-grade olfactory stem cells, which could be used to promote the recovery of spinal cord trauma, hearing loss, Parkinson's disease, amnesia, and peripheral nerve injury (Jaloux et al.). Mesenchymal cells derived from the respiratory epithelium of the middle or superior turbinates are very similar to the cells in the embryonic brain (Tung et al., 2023), further supporting the idea of the important role of mesenchymal cells in brain disorders.

These compelling indications of the similar nature of cells in the ON with those in the brain make it possible to use the ON cells as a proxy of brain cells to explore the pharmacological properties of drugs and their effects on the pathways and biological properties of the neuronal cells of the patient. In this special topic, Mihaljevic et al. utilized olfactory neuronal cells derived from biopsies of living patients with psychosis, combined with neurocognitive assessments from these same patients, to examine the clinical potential of drug targets suggested by a genome-wide association study.

Studies of the ON as a model system of brain development and neurodegeneration have shown impressive evolution, accommodating new technologies, such as epigenetic methodologies (Rhie et al., 2018) and single-cell transcriptomics (Oliva et al., 2022; Tung et al., 2023), and they are growing in scale (Evgrafov et al., 2020). This topic demonstrates increased diversity and new dimensionalities, widening the borders of cell models to use them for drug discovery (Mihaljevic et al.) and for cell therapy (Jaloux et al.) as well as continuing the addition of conditions that could be modeled by cells from the ON (Fang et al.). These studies are based on our improved understanding of the relationships between the ON and the brain (Palominos et al.; Tung et al., 2023).

The progress in this field so far lacks common protocols, therefore complicating the comparison and verification of results in different studies, which is especially important when working with heterogeneous biological samples. The iPSC model system is an example of how such problems could be overcome by closer collaboration and communication. We hope that this topic will serve as a trigger for consolidation and stimulate the advancing of this field, encouraging the exploration of cellular models even further—for example, using such tools as CRISPR and organoids.

While iPSC is a popular cellular model of brain diseases, cells derived from the ON have some advantages that make them an attractive complementary model system and contribute to their usability in both basic neuroscience and translational psychiatry.

## Author contributions

Both authors listed have made a substantial, direct, and intellectual contribution to the work and approved it for publication.

## References

[B1] Delorme B. Nivet E. Gaillard J. Häupl T. Ringe J. Devèze A.. (2010). The human nose harbors a niche of olfactory ectomesenchymal stem cells displaying neurogenic and osteogenic properties. Stem Cells Dev. 19, 853–866. 10.1089/scd.2009.0267 19905894

[B2] Etyemez S. Narita Z. Mihaljevic M. Coughlin J. M. Nestadt G. Nucifora F. C. J.. (2022). Brain regions associated with olfactory dysfunction in first episode psychosis patients. World J. Biol. Psychiatry. 24, 1–9. 10.1080/15622975.2022.2082526 35678361 PMC10503825

[B3] Etyemez S. Narita Z. Mihaljevic M. Ishizuka K. Kamath V. Yang K.. (2021). Olfactory dysfunction and face processing of social cognition in first-episode psychosis. Neurosci. Res. 176, 79–84. 10.1016/j.neures.2021.10.003 34655664

[B4] Evgrafov O. V. Armoskus C. Wrobel B. B. Spitsyna V. N. Souaiaia T. Herstein J. S.. (2020). Gene expression in patient-derived neural progenitors implicates WNT5A signaling in the etiology of schizophrenia. Biol. Psychiatry 88, 236–247. 10.1016/j.biopsych.2020.01.005 32143829 PMC10947993

[B5] Graziadei P. P. C. Graziadei G. A. M. (1979). Neurogenesis and neuron regeneration in the olfactory system of mammals. I. Morphological aspects of differentiation and structural organization of the olfactory sensory neurons. J. Neurocytol. 8, 1–18. 10.1007/BF01206454 438867

[B6] Hasegawa Y. Ma M. Sawa A. Lane A. P. Kamiya A. (2022). Olfactory impairment in psychiatric disorders: does nasal inflammation impact disease psychophysiology? Transl. Psychiatry 12, 314. 10.1038/s41398-022-02081-y 35927242 PMC9352903

[B7] Hasegawa Y. Namkung H. Smith A. Sakamoto S. Zhu X. Ishizuka K.. (2021). Causal impact of local inflammation in the nasal cavity on higher brain function and cognition. Neurosci. Res. 172, 110–115. 10.1016/j.neures.2021.04.009 33932551 PMC10693917

[B8] Kamath V. Lasutschinkow P. Ishizuka K. Sawa A. (2018). Olfactory functioning in first-episode psychosis. Schizophr. Bull. 44, 672–680. 10.1093/schbul/sbx107 28981913 PMC5890458

[B9] Kano S. Colantuoni C. Han F. Zhou Z. Yuan Q. Wilson A.. (2013). Genome-wide profiling of multiple histone methylations in olfactory cells: further implications for cellular susceptibility to oxidative stress in schizophrenia. Mol. Psychiatry 18, 740–742. 10.1038/mp.2012.120 22925834 PMC3582810

[B10] Lavoie J. Sawa A. Ishizuka K. (2017). Application of olfactory tissue and its neural progenitors to schizophrenia and psychiatric research. Curr. Opin. Psychiatry 30, 176–183. 10.1097/YCO.0000000000000327 28333692 PMC5471112

[B11] Mackay-Sim A. (2012). Concise review: patient-derived olfactory stem cells: new models for brain diseases. Stem Cells 30, 2361–2365. 10.1002/stem.1220 22961669

[B12] Moberg P. J. Kamath V. Marchetto D. M. Calkins M. E. Doty R. L. Hahn C.-G.. (2014). Meta-analysis of olfactory function in schizophrenia, first-degree family members, and youths at-risk for psychosis. Schizophr. Bull. 40, 50–59. 10.1093/schbul/sbt049 23641047 PMC3885295

[B13] Mor E. Kano S.-I. Colantuoni C. Sawa A. Navon R. Shomron N. (2013). MicroRNA-382 expression is elevated in the olfactory neuroepithelium of schizophrenia patients. Neurobiol. Dis. 55, 1–10. 10.1016/j.nbd.2013.03.011 23542694 PMC3959166

[B14] Murphy C. (2019). Olfactory and other sensory impairments in Alzheimer disease. Nat. Rev. Neurol. 15, 11–24. 10.1038/s41582-018-0097-5 30532084

[B15] Namkung H. Yukitake H. Fukudome D. Lee B. J. Tian M. Ursini G.. (2023). The miR-124-AMPAR pathway connects polygenic risks with behavioral changes shared between schizophrenia and bipolar disorder. Neuron 111 220–235.e9. 10.1016/j.neuron.2022.10.031 36379214 PMC10183200

[B16] Oliva A. D. Gupta R. Issa K. Abi Hachem R. Jang D. W. Wellford S. A.. (2022). Aging-related olfactory loss is associated with olfactory stem cell transcriptional alterations in humans. J. Clin. Invest. 132, e155506. 10.1172/JCI155506 34990409 PMC8843745

[B17] Rantanen L. M. Bitar M. Lampinen R. Stewart R. Quek H. Oikari L. E.. (2022). An Alzheimer's disease patient-derived olfactory stem cell model identifies gene expression changes associated with cognition. Cells 11, 3258. 10.3390/cells11203258 36291125 PMC9601087

[B18] Rhie S. K. Schreiner S. Witt H. Armoskus C. Lay F. D. Camarena A.. (2018). Using 3D epigenomic maps of primary olfactory neuronal cells from living individuals to understand gene regulation. Sci. Adv. 4, eaav8550. 10.1126/sciadv.aav8550 30555922 PMC6292713

[B19] Tung V. S. K. Mathews F. Boruk M. Suppa G. Foronjy R. Pato M.. (2023). Cultured mesenchymal cells from nasal turbinate as a cellular model of the neurodevelopmental component of schizophrenia etiology. bioRxiv 2023.03.28.534295. 10.1101/2023.03.28.534295 37895019 PMC10607243

[B20] Yang K. Hua J. Etyemez S. Paez A. Prasad N. Ishizuka K.. (2021). Volumetric alteration of olfactory bulb and immune-related molecular changes in olfactory epithelium in first episode psychosis patients. Schizophr. Res. 235, 9–11. 10.1016/j.schres.2021.07.016 34280869

